# New Parameters Based on Ground Reaction Forces for Monitoring Rehabilitation Following Tibial Fractures and Assessment of Heavily Altered Gait

**DOI:** 10.3390/s25082475

**Published:** 2025-04-15

**Authors:** Christian Wolff, Elke Warmerdam, Tim Dahmen, Tim Pohlemann, Philipp Slusallek, Bergita Ganse

**Affiliations:** 1 Agents and Simulated Reality, German Research Center for Artificial Intelligence (DFKI), 66123 Saarbrücken, Germany; 2 Saarbrücken Graduate School of Computer Science, Saarland University, 66123 Saarbrücken, Germany; 3 Innovative Implant Development (Fracture Healing), Departments and Institutes of Surgery, Saarland University, 66421 Homburg, Germany; elke.warmerdam@uni-saarland.de (E.W.); bergita.ganse@uks.eu (B.G.); 4 Computer Vision and Machine Learning, Hochschule Aalen, 73430 Aalen, Germany; 5 Department of Trauma, Hand and Reconstructive Surgery, Departments and Institutes of Surgery, Saarland University, 66421 Homburg, Germany

**Keywords:** gait cycle, gait analysis, digital medicine, wearable, smart insole, biomechanics

## Abstract

Instrumented insoles have created opportunities for patient monitoring via long-term recordings of ground reaction forces (GRFs). As the GRF curve is altered in patients after lower-extremity fracture, parameters defined on established curve landmarks often cannot be used to monitor the early rehabilitation process. We aimed to screen several new GRF curve-based parameters for suitability and hypothesized an interrelation with days after surgery. In an observational longitudinal study, data were collected from 13 patients with tibial fractures during straight walking at hospital visits using instrumented insoles. Parametrized curves were fitted and regression analyses conducted to determine the best fit, reflected in the highest R^2^-value and lowest fitting error. A Wald Test with t-distribution was employed for statistical analysis. Strides were classified as regular or non-regular, and changes in this proportion were analyzed. Among the 12 parameters analyzed, those with the highest R^2^-values were the mean force between inflection points (R^2^ = 0.715, *p* < 0.001, t_42_ = 9.89), the absolute time between inflection points (R^2^ = 0.707, *p* < 0.001, t_42_ = 9.83), and the highest overall force (R^2^ = 0.722, *p* < 0.001, t_42_ = 10.05). There was a significant increase in regular strides on both injured (R^2^ = 0.427, *p* < 0.001, t_42_ = 5.83) and healthy (R^2^ = 0.506, *p* < 0.001, t_42_ = 6.89) sides. The proposed parameters and assessment of the regular stride ratio enable new options for analyses and monitoring during rehabilitation after tibial shaft fractures. They are robust to pathologic GRF curves, can be determined independently from spatiotemporal coherence, and thus might provide advantages over established methods.

## 1. Introduction

**Figure 1:**
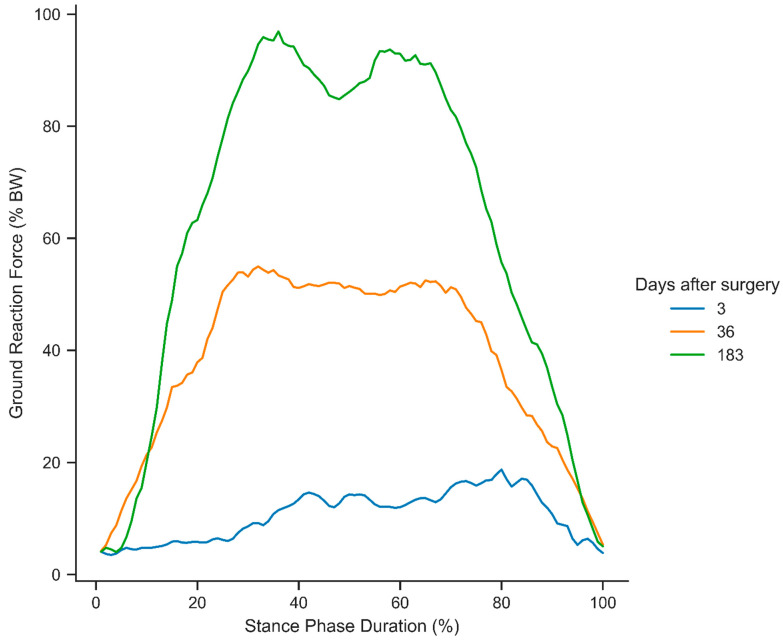
Selected stance phases of a patient during recovery from a distal tibial fracture at 3, 36, and 183 days after surgery.

Gait analysis is a fundamental tool to understand human locomotion during rehabilitation, as it provides valuable insights into biomechanical patterns and functional recovery. Motion capture and force plates are the gold standards used to record data in the laboratory. Recently, improvements in wearable technologies have introduced new devices for post-operative patient monitoring, including instrumented insoles and footwear equipped with force sensors and inertial measurement units. These have evolved into valuable tools for rehabilitation and treatment monitoring [1,2]. The devices allow for in-depth analyses of ground reaction force (GRF)-based gait parameters. Frequently used parameters describe the trajectory of the stance-phase force curve and the center of pressure (COP) [3,4]. However, these established parameters are designed for healthy gait patterns, namely, a force trajectory with two maxima and a minimum that reflect the loading and push-off phases of the gait cycle [5]. Consequently, the exact magnitudes and relative timings of these maxima and minima are parameters of interest, as they correspond to specific sections of the stance phase such as loading response or terminal stance [6]. As the foot is usually put on the ground carefully and without a rolling movement after surgery, regular parameters cannot be derived in the early stages of recovery after a tibial fracture. Further causes for the altered pattern include pain or pain avoidance, load instructions, the use of walking aids, and reduced limb functionality [7]. Figure 1 provides an example of characteristic changes in the gait cycle throughout recovery.

The analysis functions of commercial solutions frequently fall short in analyzing data from patients with lower-limb injuries. Inbuilt stride detection fails to identify the gait events of patients, as it relies on predefined healthy gait behaviors or machine learning models fitted to healthy gait patterns. We therefore propose novel parameters that are less reliant on the M-shape of the GRF. In addition, a broader stride detection method to accommodate the varied gait patterns exhibited by patients during the rehabilitation phase is suggested.

## 2. Methods

### 2.1. Study Design

In this observational prospective cohort study, all patients presenting to Saarland University Hospital with a tibial fracture between February 2022 and June 2023 were screened for eligibility to participate. Inclusion criteria were an age of 18 years and older, and a newly obtained fracture of the tibia. Exclusion criteria were further major injuries of the lower extremities, the use of walking aids and/or immobility prior to the fracture, pregnancy, inability to give consent, and participation in another ongoing clinical study within the preceding month.

Measurements were conducted in the laboratory during inpatient stays and when outpatient clinic appointments were scheduled anyway to guarantee that the frequency of consultations and degree of care were not affected. To determine whether healing occurred with or without delay, a specialist orthopedic surgeon evaluated if the fracture had healed. Instrumented pedography insoles (OpenGo insoles, Moticon GmbH, Munich, Germany) with 16 pressure sensors were fitted to the shoe size and calibrated to the body weight using the procedure provided by the recording app. Data were recorded from both feet at 100 Hz and exported using the provided software (OpenGo version 03.05.00_11808, Moticon GmbH, Munich, Germany). At each visit to the hospital, data were recorded during a 9 m straight walk. Measurements were usually conducted shortly after the surgery, around six weeks, three months, and six months post-operatively.

### 2.2. Data Processing

The extraction of stance-phase parameters from gait data entails the following key challenges: (1) sensor noise and (2) pathological stride patterns. We addressed noise by a low-pass filtering and interpolation procedure described below. To address the deformed gait shape, we replaced conventionally used landmarks with landmarks more robust to pathological gait patterns.

#### Filtering and Interpolation

First, we extracted the stance phases within the gait cycles from the time-series data. Stance phases were defined by periods of consecutive force readings exceeding 30 N with a tolerance of up to three missing values due to device faults. Activities with a duration of less than 300 ms or exceeding 3500 ms were excluded as non-stride events. A Gaussian filter (σ = 4) was applied to mitigate sensor noise and jitter.

To ensure cross-subject comparability, normalization was applied as follows: Force readings were transformed to percentage body weight. The absence of a fixed cadence leads to varying amounts of samples per stance phase. Therefore, the data were resampled using natural cubic spline interpolation to 100 equidistant samples.

All further shape-based properties, such as the indices of local maxima or inflection points for the determination of ${TP}_{1}$ and ${TP}_{2}$, were calculated based on the normalized and filtered signal. These indices were then re-applied to the unfiltered, interpolated, and normalized signal to determine force values to avoid over-smoothing.

### 2.3. Landmarks Robust to Pathological Gait Patterns

Using the first and second local maxima is the common method to determine the interval of interest on the typical M-shape. However, gait impairments make a clear distinction of maxima inconsistent. We found that the first inflection point reliably marks a point of load-bearing commitment, akin to the 80% max-force definition by Larsen et al. [3]. On the filtered and interpolated signal, this point is quite insensitive against low-force loading-phase variations.

When dealing with regular-shaped gait patterns, the ratio between the two maxima and the difference of maxima from minima can be used to describe rolling behavior during COP transmission [8] or slope [9]. For patients, this ratio is known to increase during recovery, as the stance phase develops from a plateau-like shape toward the typical M-shape [10]. Trendlines through the mid-section of the stance phase and the associated mean error calculations provide a metric for this ‘de-plateauization’ without requiring further shape assumptions. For each stance phase, it was determined whether a regular M-shape curve could be found. The M-shape requires the existence of two unambiguous local maxima with a single local minimum in between, which was often not the case with patient data. In case the result did not meet the criteria after applying the Gaussian filter, multiple strategies were applied: (i) selection of the highest/lowest candidate in case of multiple extrema occurring within 5% of overall time span, or (ii) if one candidate exceeded the others by a factor of 1.05 within the same half of the stance phase; (iii) check for signal monotony for 5% of the overall duration in both directions and eliminate candidates failing this check. For any stance phase still failing to meet the criteria, it was determined that the M-curve could not be found.

The stance phases were annotated as ‘regular’ if the M-curve could be found unequivocally, or ‘non-regular’ otherwise. We calculated the ratio of regular and non-regular stance phases for each measurement. Following this, the parameters were determined as described below.

**Figure 2:**
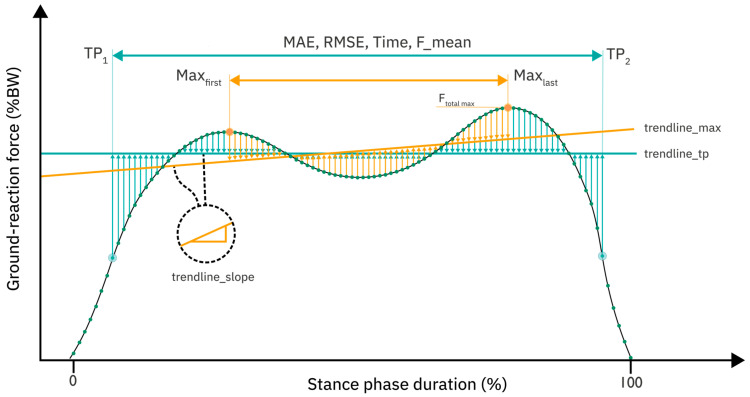
Definition of concepts which are used in many of the 12 investigated parameters. The graph shows ground reaction forces in percent body weight over the normalized stance-phase duration. Hereby, Max_first_, corresponding to F_z_2 in [3] given a regular stance-phase curve, is the first local maximum. Similarly, Max_last_, corresponding to F_z_4, is the last local maximum. Inter_max is defined as the interval from Max_first_ to Max_last_. ${TP}_{1}$ and ${TP}_{2}$ are the first and last inflection points. Inter_TP is the interval from ${TP}_{1}$ to ${TP}_{2}$. Trendline_max is the least squares regression line of the data in the interval Inter_max, shown in orange. Trendline_TP is defined similarly on the interval Inter_TP and shown in light blue. The slope of either trendline is given as trendline_slope. MAE is the mean absolute error between the trendline and the data; similarly, RMSE is the root mean square error between the trendline and the data. The concepts are used as definitory suffixes in the naming convention in Table 1.

Table 1 shows the new parameters subject to our investigation and their definitions. Hereby, ‘enclosing turning points’ are defined as the first and last instant where the second derivative of the ground reaction force curve changes sign, corresponding to inflection points (zeros of the 2nd derivative) on a discrete signal. Figure 2 provides a graphical illustration of the concepts used in this parameter design.

Our findings for the highest singular force measurement over the entire load event (F_total_max) are included for reference, because it has no shape prerequisites and is computationally trivial. Therefore, it can be determined with even the most basic baropodometric setups and wearable products. Maximum voluntary weight-bearing has been used in clinical studies regarding stroke recovery [11], to evaluate distal tibial fracture [12], and total hip arthroplasty recovery [13]. However, with the lower recording frequencies of insoles compared to treadmills, there is an increased chance of missing the true force maximum [14].

#### Parameter Analysis

All parameter data were aggregated per measurement and leg side (injured/non-injured) using the arithmetic mean. Due to the computation requirements of all inter_max-based parameters (F_trendline_max_slope, Time_inter_max, Time_inter_max_normalized), they were only determined for regular strides as well as non-regular strides with more than one maximum, but omitted for non-regular strides featuring a single maximum. Preliminary investigation revealed vast differences in means between patients, which is why Min-Max normalization was applied to facilitate between-subject comparability, using the within-subject minimum and maximum values over all the measurements accordingly.

### 2.4. Statistics

We hypothesize an interrelation between the introduced, robust parameters and the time spent in recovery. However, any single parameter will likely be insufficient to form a predictive model, as previous studies have shown considerable variability even after including additional biometric factors for gait parameters in healthy subjects [4]. To determine the best function for the interrelation between each new parameter and the time passed after surgery, the following types of functions were tested: linear, polynomial (second and third degree), and logarithmic. First, a parametrized curve was fitted for each relation, using a least squares approach. To eliminate obvious mismatches, the fitting algorithm was capped at 500 attempts of parameter alterations and the relation was discarded from further investigation in case an optimal parameter set yielding the lowest least squares curve fit could not be found within those attempts. For the remaining instances, a linear least squares regression analysis was performed on the parameter data. These data were transformed according to the respective relation (if non-linear), with the parameter value as the dependent variable and the recovery time in days after surgery as the independent variable. A slope of zero was assumed as the null hypothesis, and a Wald Test with a t-distribution of the test statistic was used to determine the *p* value of this hypothesis test. Non-significant results (*p* ≥ 0.05) were discarded. From all significant results, the best-fitting relation for each parameter–side pair was determined by selecting for the lowest fitting error based on the optimized curve and coefficient of determination.

Trial Registration: This study was registered in the German Clinical Trials Register (DRKS-ID: DRKS00025108). Ethical approval was obtained from the IRB of Saarland Medical Board (Ärztekammer des Saarlandes, Germany, application number 30/21).

## 3. Results

### 3.1. Patients

Overall, 13 patients with tibial fractures participated in this study, with repeated laboratory assessments occurring from three to seven times in the period between 3 and 175 days after surgery. Radiographic imaging confirmed that all patients showed union of the tibial fracture, as confirmed by a specialist orthopedic surgeon based on the clinical and radiographic findings. Mean demographics are shown in Table 2.

### 3.2. Stride Ratio Analysis

**Figure 3:**
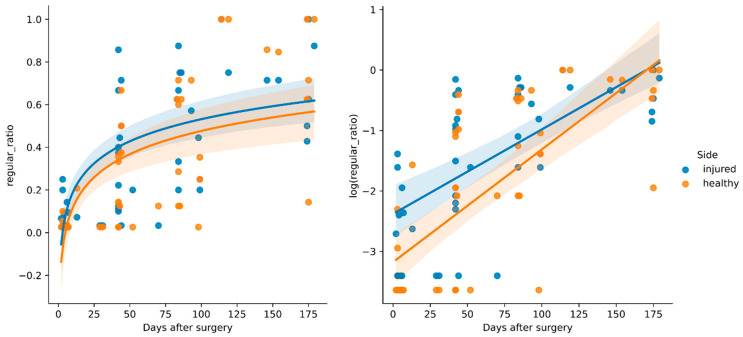
Regression results for regular_ratio, which reflects the ratio of healthy- and injured-side ground reaction force curves.

We used linear regression on the log-transformed ratio of regular strides (regular_ratio) to test whether post-operative days significantly predicted the parameter availability on the injured and healthy sides. The fitted regression models were log(regular_ratio_healthy_) = −3.18 + 0.02 days_after_surgery (*R*^2^ = 0.506, *p* < 0.001, *t*_42_ = 6.89) and log(regular_ratio_injured_) = −2.38 + 0.014 days_after_surgery (*R*^2^ = 0.427, *p* < 0.001, *t*_42_ = 5.83). The results are depicted in Figure 3. While a solid recovery trend was shown, there were multiple instances of outliers producing few or no regular steps at all throughout most of the rehabilitation phase, whereas only a few instances with a 100% regular ratio existed. While three patients did not show any regular steps in the first week after surgery, the other patients had a low ratio (mean of 0.07; SD of 0.04). Over the course of the entire study, only about a third of all steps recorded could be considered regular (mean of 0.34; SD of 0.30).

### 3.3. Parameter Analysis

**Figure 4:**
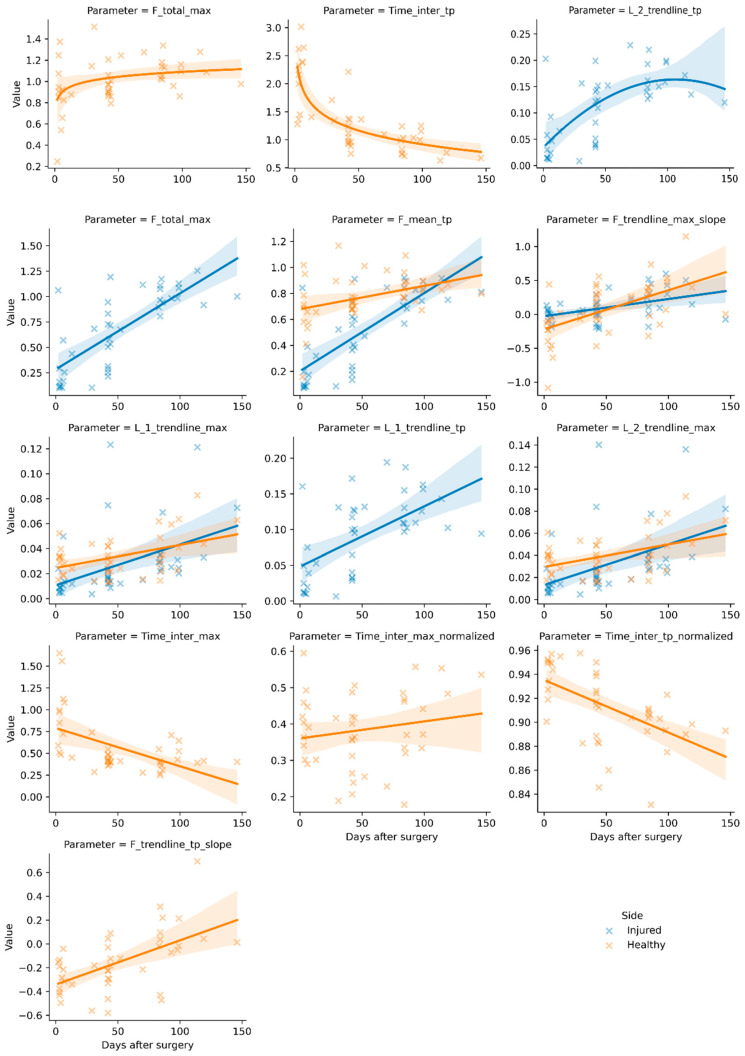
Significant (*p* < 0.05) regression results with 95% confidence interval by side and parameter over days after surgery. Force values are given in proportion to body weight, time values in seconds, or in proportion to stride length (normalized). Error metric-based parameters are noted as L_1 (MAE) and L_2 (RMSE) with suffixes referencing the trendline as per Figure 2, with their value given in proportion to body weight. Slope parameters are shown as proportion of body weight/proportion of stride length. Graphs are presented for side–parameter combinations with significant results (linear, quadratic, or logarithmic) only.

Table 3 shows the results of the parameter analysis, their relations, and the coefficients of determination. Data are only shown if the respective parameter–side pair yielded significant results. All parameters had at least one significant fitting relation to post-operative days for at least one side. The distinction between the injured and healthy sides not only led to notable differences in *R*^2^ in some cases but was also essential for the correlation of some parameters. These were L_1__trendline_TP and Gradient_loading_slope, which only showed a significant correlation with days after surgery on the injured side, and F_trendline_TP_slope, Timer_inter_TP, Time_inter_TP_normalized, Time_inter_max, and Time_inter_max_normalized on the healthy side. Figure 4 presents these regression results graphically with a 95% confidence interval. Each subfigure features one parameter. Depending on the side-specific results listed in Table 3, either one or both sides are shown for a given parameter–relation pairing.

## 4. Discussion

The present study used longitudinal insole-derived gait data from thirteen patients with tibial fractures to determine the suitability of new parameters for studying recovery in cases where the force curve of the stance phase is not regular. The most suitable parameters were the highest overall force (injured), the mean force, and the time between enclosing turning points (healthy). Several of the newly proposed parameters proved suitable when analyzing gait data from patients with tibial fractures throughout the rehabilitation process. However, most of the significant parameters were associated with low R^2^-values. We therefore recommend also considering the R^2^-value when determining the suitability of new parameters in a simple regression model.

The problem addressed in this study is that conventional parameters, such as parameters derived from the two maxima and the minimum in the classical M-shape of the curve, cannot be used when these extrema are absent or ambiguous. The first maximum is connected to the loading of the foot, followed by the in-between minimum. The second maximum reflects the forceful push-off before the foot leaves the ground. As most parameters could not be determined when the extrema were not present, there were limitations in extracting insight from force-curve data for rehabilitation research. Previous studies have analyzed parameters computed from the curve of healthy participants [4,15]. In addition, simple asymmetry parameters have been found helpful in studying rehabilitation after lower-leg fractures [10]. From a clinical perspective, it would be beneficial if, in addition to radiography-based imaging, which shows the healing progress only with a time delay and is associated with radiation exposure, gait analyses could be used to monitor recovery after injuries in a timelier manner. The early diagnosis of healing delays could allow earlier intervention [16]. Several recent studies have dealt with the use of wearables to continuously monitor recovery in the daily life of patients [17,18]. Advances in the usability of instrumented insoles and their increasing recording frequencies have opened up new possibilities for much timelier and more individual, patient-centered care. Further studies now need to evaluate the suitability of the new parameters in other patient groups with lower-limb injuries or disorders, and for the prediction of complications, with further endpoints.

### 4.1. Stride Quality

The results of the stride ratio analysis showed a considerable proportion of non-regular strides during the early stages of recovery. Even in the later phases of rehabilitation, it was only possible to achieve a regular ratio of 100% in a few cases. This is not necessarily an indication of a lack of recovery. In previous studies using the same insoles under laboratory conditions, healthy participants reached about 80% regular strides without the use of further data refinement strategies [4]. This finding underlines the necessity for gait parameters, which are independent of idealized force-graph shapes. Our regression analysis showed an association of a gain of regularity with recovery time.

### 4.2. Parameters

All proposed parameters correlated with days after surgery. However, some of them had low variability and thus need to be handled with more caution. The side has a relevant impact on healing patients [18]. For this reason, stride analysis should be performed sidewise instead of with aggregates over all instances. Pooling GRF data of both sides may still yield significant results, but model quality will likely decrease. Furthermore, the one-sidedness of some parameters enables one-sided monitoring. Since there are several instances of only the healthy side showing a significant correlation, it would also be feasible to only measure this side during recovery. To reduce the risk of complications, i.e., delayed union or non-union, multiple devices and concepts are in development that aim to provide monitoring, live patient feedback, and means of active intervention through actuation [16]. When measuring forces through a smart implant with sensing and acting capabilities, this implant will only be placed in the injured leg, which makes it of interest to identify parameters that will work in this case. In relation to this, the highest model quality was found for the reference measure F_total_max on the injured side. This finding shows that rehabilitation monitoring using a single insole and a very basic baropodometric setup already provides valuable data for the clinician. Furthermore, it underlines the importance of side separation during analysis, as the F_total_max (healthy) model provides the weakest results out of all significant models.

The model quality varied notably between parameters and sides. While almost all significant parameter–side pairings yielded very strong correlations with recovery time, the coefficients of determination (R^2^) ranged between 0.141 and 0.722 with only a few reaching above 0.5. This is an indication that for a low R^2^, in most cases, the model in question lacks additional independent variables. In order to produce a model with predictive capability beyond just correlation, these factors would need to be identified and included. This hypothesis is supported by the findings of Hollman et al. [19], in which several biometric factors influence gait patterns to a point where established methods exist to identify individuals based on GRF features [20]. These influences persist even after weight/time normalization, although their impact is decreased [4,21,22]. A concurrent explanation is the use of Min-Max scaling in our study, which normalizes features to the range [0,1]. This approach was chosen to enable between-subject comparison and trend analysis. However, the difficulty of curve fitting might be increased since Min-Max scaling is sensitive to outliers, which reduces the overall R^2^. When monitoring the progression of a patient in the clinic, this Min-Max scaling is not necessary for a within-subject comparison.

In their general trends, most parameters described predicted behavioral changes during recovery. The increase in error metric-based parameters (L_1_, L_2_) indicated an increase in variance during the central parts of the stance phase, which can be explained as a ‘de-plateauization’ of the gait pattern, e.g., development toward the distinct M-shape. This is supported by the decrease in Time_inter_max and increase in Time_inter_max normalized to the healthy side; the overall stride becomes quicker, and the maxima become more pronounced, as the functional deficiency of the injured leg decreases [10]. This effect also occurs in F_trendline_TP_slope (healthy), as the increase in the regression slope is caused by a relative increase in the push-off phase. The continuous decrease in loading and unloading slope gradients points toward a smoother unreeling behavior with less relative impact shock, marking a return to natural heel-to-toe movement.

To the best of the authors’ knowledge, there are no other publications dealing with a longitudinal parameter analysis of the GRF stance-phase curve specifically for altered gait using insoles. Likewise, there has been related work on gait parameter derivation from insole GRF data in patients with hemiplegia who vary distinctly in the plantar pressure difference (PPD) between the feet [23]. The PPD and phase coordination index (PCI), which determine motion asymmetry based on stride time differences, also distinguished between stroke survivors and healthy individuals [24]. The PCI has further applications in the analysis of geriatric patients and patients with Parkinson’s [25,26]. This approach was refined and new additional indices were introduced to increase differentiation rates between healthy adults and stroke patients [27]. A natural limitation of asymmetry parameters such as PPD, PCI, and partially COP is the need for (spatio)temporal coherence. Therefore, one-sided recording or the analysis of isolated, individual strides outside the context of a walking bout is either impossible or non-sensical. This also applies to data contaminated by temporal sensor drift, which is an inherent challenge of insole systems [28]. While sensor drift has a negligible impact in a short-term laboratory setting, it is a factor in long-term monitoring scenarios.

### 4.3. Correlation with Bone Healing

Fracture union in the present study was determined by an experienced specialist orthopedic surgeon based on the clinical and radiographic findings combined. As all patients healed quickly, this was considered sufficient for the present research question. Scientifically, however, a quantitative method is desirable to correlate gait parameters with healing progress. Currently, the availability of objective outcome parameters to quantify the progress of fracture healing in patients is insufficient. Out of the few alternatives applicable to human patients, the RUST score is among the most popular measures [29]. Based on X-ray images, it considers whether the cortical bone has reached radiographic continuity. However, an increase in calcification does not directly correlate with increases in fracture stiffness [30]. In addition, radiographs would need to be taken at defined time points to allow standardization and comparability in such a study, which would be associated with additional exposure to ionizing radiation [31]. Computed tomography imaging is associated with even higher radiation doses and therefore not an option. Magnetic Resonance Imaging (MRI) is problematic in patients with metal implants due to artifacts and therefore not an option either. Among the most promising emerging methods to study fracture healing progress in vivo are Laser-Doppler and white-light spectroscopy [32] as well as photoacoustic imaging [33].

### 4.4. Limitations

There are several limitations to this study. The sample size decreased toward the later time points. This patient number is the result of the single-center study design implemented in a busy university hospital. Future studies may favor a multi-center study design. The low patient number might have exacerbated the high variability found in the data, which influences not only model quality but also the process of relation fitting. Furthermore, it might be biased regarding both the rate and quality of the changes in gait features over time in comparison to the general population. Although none of the patients showed any complications, the usage of days after surgery as a time metric is prone to inter-subject variability and might not be equally representative of a gain of function in the affected limb.

### 4.5. Further Applications

The findings of the present study are not only of relevance for patients with tibial fractures but may also be useful for gait analyses in people with disabilities and neurological disorders, i.e., cerebral palsy [34]. Animals with hooves, such as horses, generally do not have an M-shaped GRF curve during the stance phase but usually show only one maximum [35]. Similar findings were obtained for rabbits [36]. The new parameters may be of use in analyzing gait in such animals.

Insoles facilitate studies conducted in real-world settings, enabling the measurement of patients without supervision and within realistic environments. Periodic data transmission allows for continuous monitoring over typical recovery periods lasting several weeks, providing unprecedented insights into a patient’s gait development [18]. However, this cutting-edge approach also presents new challenges. Individuals in their daily lives, regardless of any handicap, generate a variety of load-bearing non-gait motion data that must either be distinguished from regular gait data or be analyzed separately for potential effects on recovery. Conceptually, this presents a challenge for the implementation of stride detection algorithms that do not rely on additional information typically available in a laboratory setting, such as video recordings or strict study designs: they must be robust enough to detect wide varieties of strides without becoming prone to a possibly greater variety of false positives. One potential solution is to employ machine learning algorithms in various forms [37,38,39]. In the long term, a system taking into consideration overall gait behavior, development over time, and lifestyle factors could permit prediction and offer individualized feedback to patients [40,41]. In the short term, a live event classification system could serve as a foundation for more complex prediction algorithms. However, this approach faces a bootstrapping problem: the data required to train such a system—extensively annotated GRF gait recordings—are the same type of data the system is meant to produce. The substantial increase in data collected through near-continuous measurement compared to isolated trials in a gait lab makes manual annotation impractical. To the best of the authors’ knowledge, there is no existing data repository that meets the essential criteria both qualitatively and quantitatively. Nevertheless, to develop a meaningful machine learning application on a medium-to-large scale, robust analysis and therefore annotation at the single-event level will be a likely prerequisite.

## 5. Conclusions

When using instrumented insoles to study the rehabilitation progress after tibial fractures, classical parameters derived from the ground reaction force curve often cannot be used to monitor early changes. We proposed new parameters applicable to individuals in the early stages of recovery after a tibial fracture and hypothesized interrelation with days after surgery. Among the newly proposed parameters, the mean force between enclosing turning points, the absolute time between enclosing turning points, and the highest overall force appeared most suitable. The proposed parameters and the approach to assess the number of regular strides enable new options for analyses and monitoring in rehabilitation after tibial shaft fractures. Further studies might be able to formulate advanced models that allow more explicit conclusions in relation to the process of healing or detection of complications.

## Figures and Tables

**Table 1:** Names, definitions, computation equations, and units of the parameters investigated in this work. For a stride given as an array of 100 equidistant force measurements [y_1_, y_2_, …, y_100_], iMax_first_, iMax_last_, iTP_1_, and iTP_2_ denote the array indices of the first/last maximum and TP_1/2_, respectively. MAE($\hat{y})$ and RMSE($\hat{y})$ are defined as the mean average error and root mean square error of a given least squares regression line $\hat{y}={\hat{\beta }}_{1}t+{\hat{\beta }}_{0}$. The function abs(t) returns the absolute timestamp of measurement y_t_ at array index t.

Parameter Name	Definition	Computation	Unit
F_mean_TP	Mean force between enclosing turning points	$\frac{\sum_{{i=iTP}_{1}}^{i{TP}_{2}}y_{i}}{{iTP}_{2}-{iTP}_{1}}$	%Bodyweight
F_total_max	Highest overall force measurement in the entire stance phase	${max}_{1\leq i\leq 100}\;y_{i}$	%Bodyweight
F_trendline_max_slope	Slope of the least squares regression line ${\hat{y}}_{max}={\hat{\beta }}_{1}t+{\hat{\beta }}_{0}$ of force readings between the first and the last maxima ^1^	$\begin{array}{l} {\hat{\beta }}_{1}=\frac{{SS}_{ty}}{{SS}_{tt}},\\ {iMax}_{first}\leq t\leq {iMax}_{last} \end{array}$	$\frac{\%Bodyweight}{\%Stance\;Duration}$
F_trendline_TP_slope	Slope of the least squares regression line ${\hat{y}}_{tp}={\hat{\beta }}_{1}t+{\hat{\beta }}_{0}\;$of all force readings between enclosing turning points	$\begin{array}{l} {\hat{\beta }}_{1}=\frac{{SS}_{ty}}{{SS}_{tt}},\\ i{TP}_{1}\leq t\leq {iTP}_{2} \end{array}$	$\frac{\%Bodyweight}{\%Stance\;Duration}$
L_1__trendline_max	Mean average error of the least squares regression line corresponding to F_trendline_max_slope	$MAE({\hat{y}}_{max})$	%Bodyweight
L_1__trendline_TP	Mean error of the least squares regression line corresponding to F_trendline_TP_slope	$MAE({\hat{y}}_{tp})$	%Bodyweight
L_2__trendline_max	Root mean square error of the least squares regression line corresponding to F_trendline_max_slope	$RMSE({\hat{y}}_{max})$	%Bodyweight
L_2__trendline_TP	Root mean square error of the least squares regression line corresponding to F_trendline_TP_slope	$RMSE({\hat{y}}_{tp})$	%Bodyweight
Time_inter_max	Absolute time passed between the first and last local maxima ^1^	$abs\left({iMax}_{first}\right)-abs(i{Max}_{last})$	Seconds
Time_inter_max_normalized	Time passed between the first and last local maxima in proportion to overall step duration ^1^	$\frac{{iMax}_{first}-{iMax}_{last}}{100}$	×100%Stance Duration
Time_inter_TP	Absolute time passed between enclosing turning points in seconds	$abs\left({iTP}_{1}\right)-abs({iTP}_{2})$	Seconds
Time_inter_TP_normalized	Time passed between enclosing turning points in proportion to overall step duration	$\frac{{iTP}_{1}-{iTP}_{2}}{100}$	×100%Stance Duration

^1^ This parameter can only be calculated if more than one local maximum is present within the stance-phase curve and is omitted otherwise.

**Table 2:** Demographics of the subject group.

N (male/female)	13 (5/8)
Age [y]	49 ± 14
Weight [kg]	75 ± 12
Body height [cm]	175 ± 10
Side injured (r/l)	(4/9)

**Table 3:** List of parameters that showed a significant (*p* < 0.05) correlation with days post-op with corresponding relation type, coefficient of determination, *p* value, and test value given as t_value (df). R^2^ values above 0.7 and *p* values below 0.001 are presented in italics.

Parameter	Side	Relation	R^2^	*p* Value	*t*-Test (df)
F_mean_TP	Healthy	Linear	0.381	*<0.001*	4.96 (42)
F_mean_TP	Injured	Linear	*0.715*	*<0.001*	9.89 (42)
F_total_max	Healthy	Logarithmic	0.141	0.020	2.57 (42)
F_total_max	Injured	Linear	*0.722*	*<0.001*	10.05 (42)
F_trendline_max_slope	Healthy	Linear	0.269	*<0.001*	3.84 (42)
F_trendline_max_slope	Injured	Linear	0.386	*<0.001*	4.97 (42)
F_trendline_TP_slope	Healthy	Linear	0.365	*<0.001*	4.79 (42)
L_1__trendline_max	Healthy	Linear	0.255	*<0.001*	3.7 (42)
L_1__trendline_max	Injured	Linear	0.369	*<0.001*	4.78 (42)
L_1__trendline_TP	Injured	Square	0.245	0.001	3.56 (42)
L_2__trendline_max	Healthy	Linear	0.223	0.002	3.39 (42)
L_2__trendline_max	Injured	Linear	0.370	*<0.001*	4.78 (42)
L_2__trendline_TP	Injured	Square	0.253	*<0.001*	3.63 (42)
Time_inter_TP	Healthy	Logarithmic	*0.707*	*<0.001*	9.83 (42)
Time_inter_TP_normalized	Healthy	Linear	0.479	*<0.001*	6.06 (42)
Time_inter_max	Healthy	Linear	0.363	*<0.001*	4.77 (42)
Time_inter_max_normalized	Healthy	Linear	0.162	0.008	2.77 (42)

## Data Availability

The data underlying this article cannot be shared publicly due to the privacy requirements of individuals that participated in the study set by the study protocol. Derived and aggregated data will be shared on reasonable request to the corresponding author after signing a usage agreement.

## Abbreviations

COP	Center of pressure
GRF	Ground reaction force
MAE	Mean average error
PCI	Phase coordination index
PPD	Plantar pressure distribution
RMSE	Root mean square error
TP	Turning point
